# A Comparative Study on Heavy Metal Removal from CCA-Treated Wood Waste by *Yarrowia lipolytica*: Effects of Metal Stress

**DOI:** 10.3390/jof9040469

**Published:** 2023-04-13

**Authors:** Dan Xing, Sara Magdouli, Jingfa Zhang, Hassine Bouafif, Ahmed Koubaa

**Affiliations:** 1 Institut de Recherche sur les Forêts, Université du Québec en Abitibi-Témiscamingue, Rouyn-Noranda, QC J9X 5E4, Canada; dan.xing@uqat.ca; 2 Lassonde School of Engineering, York University, Toronto, ON M3J 1P3, Canada; 3 State Key Laboratory of Biobased Material and Green Papermaking, Qilu University of Technology, Shandong Academy of Sciences, Jinan 250353, China; 4 Centre Technologique des Résidus Industriels en Abitibi Témiscamingue, 433 Boulevard du Collège, Rouyn-Noranda, QC J9X 0E1, Canada

**Keywords:** bioremediation, heavy metals, *Y. lipolytica*, CCA-treated wood, copper stress

## Abstract

Bioremediation is an effective way to remove heavy metals from pollutants. This study investigated the effects of *Yarrowia lipolytica* (*Y. lipolytica*) on the bioremediation of chromated copper arsenate (CCA)-treated wood wastes. Copper ions stressed the yeast strains to improve their bioremediation efficiency. A comparison of changes in morphology, chemical composition, and metal content of CCA wood before and after bioremediation was conducted. The amount of arsenic (As), chromium (Cr), and copper (Cu) was quantified by microwave plasma atomic emission spectrometer. The results showed that yeast strains remained on the surface of CCA-treated wood after bioremediation. The morphologies of the strains changed from net to spherical because of the Cu^2+^ stress. Fourier-transform infrared spectroscopy showed that carboxylic acid groups of wood were released after removing heavy metals. A large amount of oxalic acid was observed when the optical density (OD_600nm_) was 0.05 on the 21st day. Meanwhile, the highest removal rate of Cu, As, and Cr was 82.8%, 68.3%, and 43.1%, respectively. Furthermore, the Cu removal from CCA-treated wood increased by about 20% after Cu^2+^ stress. This study showed that it is feasible to remove heavy metals from CCA-treated wood by *Y. lipolytica* without destroying the wood structure, especially by copper-induced *Y. lipolytica*.

## 1. Introduction

The quality of human life is highly dependent on the conditions of the terrestrial environment. The harmfulness of solid pollutants, such as plastic, batteries, and preservative-treated wood (PTW) wastes, attracts human attention due to their negative impacts on the environment and human health [1]. PTW has been widely used in construction, railway, boardwalk, and electricity poles by humans for many decades. However, heavy metals in wood preservatives are harmful to the environment. Previous reports show that inorganic metal salts leaching from solid pollutants are toxic and damage human health even at low concentrations [1,2]. Therefore, the decontamination of PTW is an important and urgent issue rather than being directly released into the environment.

CCA has historically been one of the most widely used waterborne preservatives for wood due to its efficiency and low cost [3]. Arsenic, copper, and chromium in the CCA preservative have a great risk to human health. They are leached into soil or water when the treated wood is exposed to the environment. Thus, the use of CCA-treated wood has been banned in Europe since 2004 [4]. Additionally, the EPA and the wood industry decided in 2003 to stop using CCA-treated wood in most residential construction because it contained arsenic. However, due to its low cost, much CCA-treated wood waste is still in use, especially in several countries (e.g., India, China, and Brazil). As a result of scientific concerns, finding an economical and feasible way to alleviate environmental contamination is significant when CCA-treated wood is at the end of its service life.

Various approaches, such as landfilling, incineration, pyrolysis, gasification, chemical extraction, electro-dialysis, and bioremediation, have been investigated to deal with CCA-treated wood wastes [5,6,7]. Among those methods, incineration is the most used method. During the incineration of CCA-treated wood, Cr and Cu compounds mostly remain in the ash, while As will be diffused in the air [8]. Therefore, the premise of incineration treatment should be equipped with an advanced filtration system. Pyrolysis is usually performed at 400–800 °C accompanied by the release of harmful traces of arsenic [9]. Worryingly, incineration and gasification will accelerate carbon emissions and are not conducive to realizing carbon neutrality goals. Landfilling may contaminate the groundwater because of the leaching potential of contaminated substances. Thus, landfilling is not a legal option in most countries. Chemical pretreatment may generate intermediate by-products and secondary pollutants even if it can efficiently remove heavy metals from CCA-treated wood. Bioremediation is considered a promising and safe method. It is also the least mature solution and requires further analysis and discussion.

Microorganisms such as fungi can secrete oxalic acids outside the cells, which is important in removing metal compounds in CCA wood. Many wood-rot fungi have been used to treat wood waste by removing toxic metals or bioconverting heavy metals into less toxic or completely harmless forms [10]. In addition to *Saccharomyces cerevisiae*, *Cryptococcus* sp., *Candida* sp. *PS33*, and *black mold* have also been studied to bioremediate CCA-treated wood wastes and pollutants [11,12,13,14,15]. Fungi absorb heavy metals by chemical transformation through redox reactions and bioaccumulation through passive diffusion, facilitated diffusion, or active transport [5]. In addition to the microbes’ biofilm’s metal tolerance, their secondary metabolites play key roles in decontamination [16]. Notably, *Y. lipolytica* and *Aspergillus niger* adsorb Pb(II), Cr(III), Cr(VI), Zn(II), Cu(II), As(V), and Ni(II) ions from aqueous solutions and successfully purify sewage [17,18]. *Y. lipolytica* is a model yeast for organic acid production, especially citric acids [19]. Moreover, the changes in different morphological forms of *Y. lipolytica* help it easily adapt to new environmental conditions [20]. Numerous studies have investigated the yeast’s response to different domesticated conditions, such as heavy metal stress, temperature, pH, nutrient availability (carbon, nitrogen, trace elements, etc.), dissolved oxygen concentration, electrochemical stress, agitation, and aeration [20,21,22]. Previous literature also found that *Y. lipolytica* is easy to domesticate using oleic acid as the oxygen vector [23]. In summary, the yeasts were more resistant to heavy metal environments after domestication.

However, studies on removing metal ions from solid contamination (e.g., CCA-treated wood) by *Y. lipolytica* are rare. Although *Y. lipolytica* effectively removes heavy metal ions from aqueous solutions, its ability to remove metals from CCA-treated wood is uncertain. Removing metal ions from aqueous environments is much easier than from CCA wood because the metals in CCA wood are bound to the wood components. It is important to reveal the mechanism of the metal removal from CCA-treated wood by *Y. lipolytica* to provide more theoretical support for the biological decontamination of CCA wood wastes.

Thus, *Y. lipolytica* was used to remove heavy metals from CCA-treated wood to investigate its decontamination properties in this study. The change in the wood structure and surficial chemicals were explored. Yeast communities are shielded from environmental stress by secreting secondary metabolites and transforming their morphology. Cu^2+^ solutions with different concentrations were used to induce *Y. lipolytica* to increase heavy metals removal further. The effect of Cu^2+^ stress on the morphology of *Y. lipolytica* and the removal rate of heavy metals were also explored. This study provides a promising potential for further application of *Y. lipolytica* for toxic metals removal from solid wastes.

## 2. Materials and Methods

### 2.1. Materials and Microorganisms

Sigma-Aldrich Canada (Oakville, ON, Canada) supplied all reagent-grade chemicals used in this study. The Global Bioresource Center provided the *Yarrowia lipolytica* 20,460 strain. The cells of activated *Y. lipolytica* were preserved at −80 °C in a mixture of glycerol and yeast malt (YM) medium. The waste CCA-treated wood used in this study came from shredded poles supplied by Tred’si (North America). They were crushed into small pieces (0.3 mm). The CCA-treated wood waste contained Cu of 2.46 mg·g^−1^, Cr of 4.54 mg·g^−1^, and As of 1.57 mg·g^−1^ before bioremediation.

### 2.2. Culture Medium and Conditions

The *Y. lipolytica* strain was first cultivated in a YM medium for 48 h. The YM medium comprised 3 g of yeast extract, 3 g of malt extract, 10 g of glucose anhydrous, 5 g of tryptic soy broth, and 1 L of deionized (DI) water. The pH of the activated strain was 3.5. The activated strain was then transferred to a growth medium and cultivated in a shaker. The growth medium contained 50 g of glucose, 0.25 g of (NH_4_)_2_SO_4_, 1.7 g of KH_2_PO_4_, 12 of NaH_2_PO_4_, 1.25 g of MgSO_4_·7H_2_O, 0.5 g of yeast extract, and 1 L of DI water. The pH of the growth medium was 4.72.

### 2.3. Metal Stress of Y. lipolytica

*Y. lipolytica* was inoculated in the growth medium, and its optical density (OD) was adjusted to 0.6. The OD value was measured using a UV-vis photo spectrometer (GEN10S, Madison, WI, USA) at an excitation wavelength of 600 nm. Growth mediums with high copper ion concentrations of 0.5, 0.6, 0.7, 0.8, and 1.0 g·L^−1^ were prepared for metal stress using CuCl_2_·2H_2_O. An inoculated growth medium without metal ions served as a control. The above medium of 100 μL was withdrawn and transferred into the solid YM medium at intervals of 1, 4, 8, 12, and 14 days to conveniently observe the growth of the strain with the naked eye. Two replicates were operated the same as above. The domesticated strains were preserved at −80 °C in glycerol medium.

### 2.4. Bioremediation Procedure

Laboratory experiments explored the effect of metal stress on the bioremediation of CCA-treated wood. The OD_600nm_ value of the growth medium in flasks was adjusted to 0.05, 0.1, 0.3, 0.6, and 0.9 by adding the activated *Y. lipolytica*. The pH of OD_600nm_ = 0.05, 0.1, 0.3, 0.6, and 0.9 was 4.7, 4.64, 4.62, 4.59, and 4.55, respectively. These strains were cultivated in flasks in a shaker at a rate of 125 rpm for 24 h at 27 °C. CCA-treated wood wastes of 5g were then placed into fermented liquid media of 200 mL. CCA wood samples treated with distilled water and growth medium served as controls. The flasks inoculated with different strain concentrations were sampled at 1, 3, 6, 9, 15, and 21 days compared to the controls. Two replicates of each sample with different OD_600nm_ values and control samples were performed. Flask liquids were dumped into 50 mL tubes to measure their pH using a pH meter (HI-2002, HANNA, Padova, Italy). The wood particles were separated from the solution through a strainer. Furthermore, the treated wood particle was washed with distilled water three times and then placed in an oven overnight at 50 °C for characterization and reuse.

The following experiment was performed with different amounts of wood sawdust instead of strain concentrations. Four different quantities of wood (3, 5, 7, and 9 g) were used, where the OD_600nm_ was 0.6, and the other conditions were kept the same. Two replicates of each sample with different amounts of CCA wood were performed.

The domesticated strains of the sample (A), (B), (C), (D), (E), and (F) were selected to perform the bioremediation tests to investigate the effects of Cu^2+^ stress on heavy metal removal from CCA-treated wood wastes (Table 1). Samples (A) and (B) were stressed by copper for 4 and 8 days, respectively, with Cu^2+^ concentrations of 600 mg·LTab^−1^. Samples (C) and (D) were stressed for 12 and 14 days, respectively, with Cu^2+^ concentrations of 700 mg·L^−1^. Samples (E) and (F) were stressed for 12 days with Cu^2+^ concentrations of 800 and 1000 mg·L^−1^, respectively. The OD_600nm_ was 0.6, and the other conditions were the same as the abovementioned parameters. The strains with OD_600nm_ = 0.6 without adding Cu^2+^ were used as a control. The growth medium without any strains was set as a blank control. All samples had two replicates.

### 2.5. Characterization of CCA-Treated Wood

After bioremediation, the surface chemical properties of the CCA-treated wood samples were analyzed using a Fourier-transform infrared spectrophotometer (IR_Tracer-100, Shimadzu, Kyoto, Japan) with an attenuated total reflection. Wood samples were scanned at wavelengths in the range of 500–4000 cm^−1^ with a resolution of 4 cm^−1^.

The surficial morphology of the wood samples was characterized using scanning electron microscopy (SEM) (SU1510, HITACHI, Kyoto, Japan) with an accelerating voltage of 10 kV. The wood residue was sputter-coated with gold powder before testing.

Organic acids produced by fungi in the solution were determined by HPLC with a Biorad Aminex HPX-87 H column (300 × 7.8 mm) (Alltech Assoc. Inc., Deerfield, IL, USA) at 40 °C using ultraviolet detection (210 nm). The results are the average of three measurements. The eluent was 5 mM H_2_SO_4_ at a flow rate of 0.6 mL·min^−1^. Liquid supernatants containing Cu and Cr were pretreated with the resin Chelex-100 (Sigma Aldrich, St. Louis, MO, USA) to prevent analytical interference by heavy metals. The tested samples were diluted with an equal volume of phosphate buffer (0.08 M, pH 6.5) and supplied with Chelex 100 (0.1 g). The mixture solution was shaken before the HPLC test and passed through a membrane filter (0.45 μm) based on previous literature [24].

The heavy metal content of wood wastes before and after bioremediation was detected to analyze the metal removal efficiency of *Y. lipolytica*. An Agilent 4100 Microwave Plasma Atomic Emission Spectrometer (Agilent Technologies, Melbourne, Australia) equipped with a standard glass concentric nebulizer and cyclonic spray chamber (Agilent Technologies, Melbourne, Australia) was used to analyze the heavy metals in the CCA-treated wood wastes before and after bioremediation. Before each test, 20 s torch stabilization and 10 s uptake were applied. Meanwhile, a 5 s read time with three replicates was used for the emission measurement of each sample. The metal content is the average of three measurements.

## 3. Results and Discussion

### 3.1. Morphology of Bioremediation Wood

**Figure 1:**
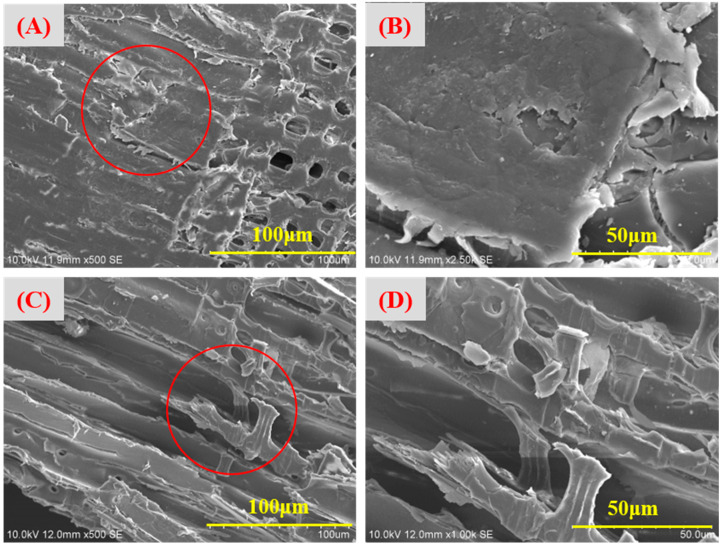
Micro-morphology of CCA-treated wood before treatment at 100 μm (**A**) and 50 μm scale (**B**); after treatment with uninoculated medium for 21 d at 100 μm (**C**) and 50 μm scale (**D**).

**Figure 2:**
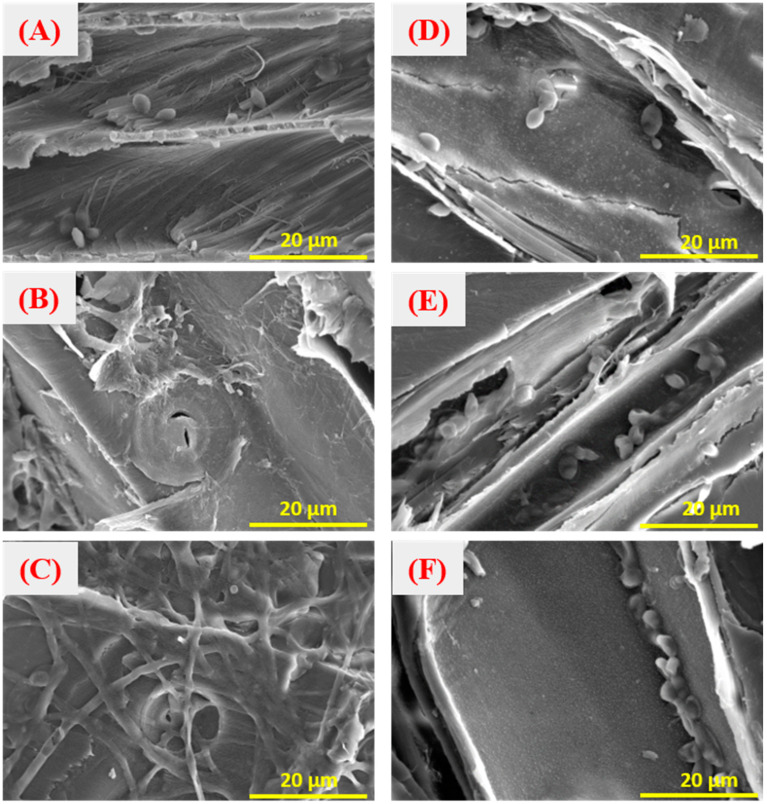
Micro-morphology of CCA-treated wood treated with the *Y. lipolytica*: CCA wood treated with OD_600nm_ = 0.05 for 1 d (**A**), 9 d (**B**), and 21 d (**C**), and CCA wood treated with domesticated strains of sample B for 1 d (**D**), 9 d (**E**), and 21 d (**F**).

The surfaces of waste CCA-treated wood displayed a relatively smooth and intact structure (Figure 1). The microbial strains and medium treatments still need to change the structure of CCA-wood, as depicted in Figure 1A,B. The original structure of the wood was preserved in CCA-treated wood after bioremediation with *Y. lipolytica*. This result contradicts previous findings where the lignin and cellulose of wood were decomposed [25]. The intact structure of wood facilitates its recycling. However, microbial strains covered the wood samples’ surface after bioremediation. Strains adhered to the CCA wood surface increased with the increase in cultivation time (Figure 2A–C). The SEM results also showed that the cells of *Y. lipolytica* adhered to CCA-treated wood were ellipsoid with bipolar budding patterns. The size of the ellipsoidal cells was around 5 μm. Meanwhile, some characteristic features of filamentous hyphae were also observed, especially for longer cultivation times.

**Figure 3:**
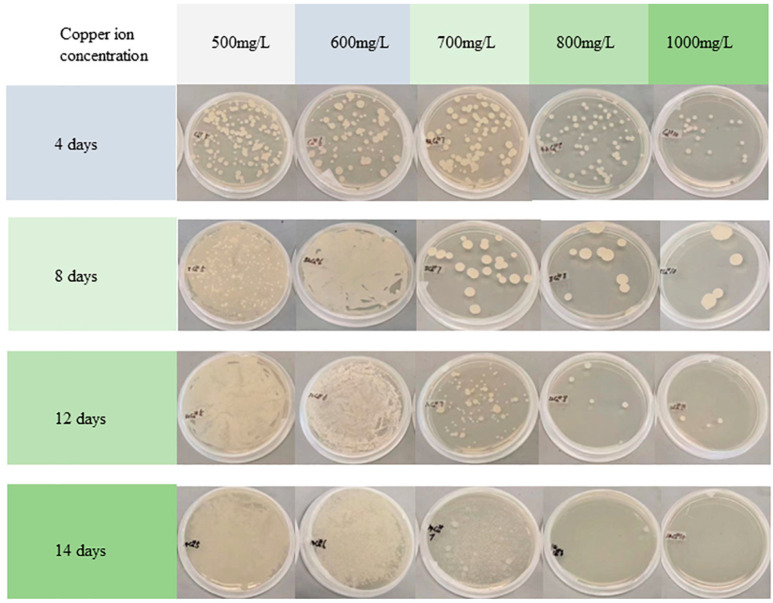
Digital photos of the growth status of the domesticated *Y. lipolytica*.

Compared to the original strains, the Cu^2+^-domesticated strains had an ellipsoidal shape and partially changed into much smaller particles (Figure 2D–F). These observations agreed with the results of digital images of the fungi, as shown in Figure 3 and Table 2. The morphological characteristics of strain (B) after Cu^2+^ stress changed from large, oval, and round cells to small ones (Figure 3). These characteristics mainly occurred when the concentration of Cu^2+^ ranged from 500 to 700 mg·L^−1^, especially with an increase in cultivation days. This result indicated that the domesticated strains with round cells and small sizes are more adaptable to higher copper ion concentrations than the original strain. A previous report drew a similar conclusion that morphological changes of *Y. lipolytica* were in response to different heavy metal stress [20]. Like the original strain, microbial remediation with the domesticated strains did not destroy the surface structure of CCA-treated wood. Furthermore, many strains grew on the surface of CCA-treated wood. This observation indicated that the changes in morphological characteristics of strains hardly change the reaction mechanism of bioremediate wood waste.

### 3.2. Surface Chemical Properties of CCA-Treated Wood Samples

**Figure 4:**
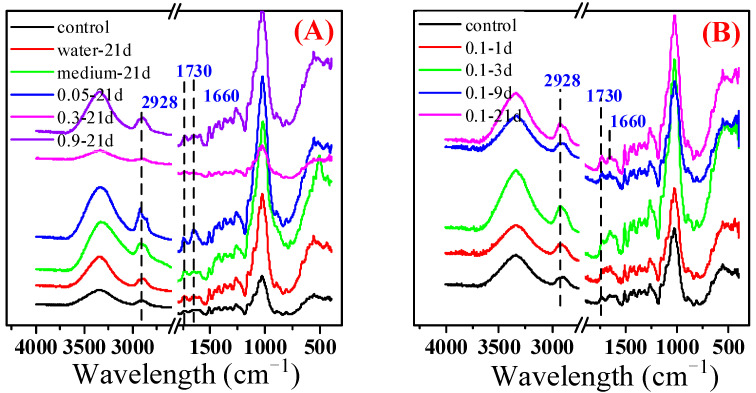
FTIR spectra of CCA wood treated with different conditions for 21 days (**A**) and with the concentrations of *Y. lipolytica* (OD_600nm_ = 0.1) for different cultivation days (**B**).

The surface chemical groups of the CCA-treated wood before and after bioremediation were characterized by FTIR, as shown in Figure 4. A broad and intensely sharp band at 3444 to 3330 cm^−1^ was observed. This band can be attributed to the stretching vibration of the hydroxyl group of bonded water in the CCA-treated wood. The asymmetric and symmetric vibrations of the C–H bonds were located at the 2922 and 2853 cm^−1^ wavenumbers, respectively. Those results demonstrated the presence of aliphatic structures in the CCA-treated wood. The peaks at 1240 and 1147 to 1031 cm^−1^ and the continuation spectra between 900 and 700 cm^−1^ were attributed to C–O, C–C, or C–OH vibrations of aromatic structures and C–H bonds of aromatic structures, respectively. The peaks at about 1650 to 1750 cm^−1^ indicated the presence of C = O vibrations. Nadaroglu et al. (2015) also observed these characteristic peaks in the FTIR spectrum of CCA-treated wood [26]. Furthermore, the wide absorption band at 1650–1730 cm^−1^ was attributed to the stretching vibrations of the C = O group in the CCA-treated wood. However, this band was split into two peaks after bioremediation. This result can be explained by copper (II) ions complexed with the acetyl or carboxylic acid bonds in the CCA-treated wood, causing a broad absorption band [27]. The copper ions in the CCA-treated wood were removed by microbial remediation with *Y. Lipolytica*, releasing carboxylic acid.

### 3.3. Bioremediation of CCA-Treated Wood

#### 3.3.1. Influence of Initial Strain Concentrations

**Figure 5:**
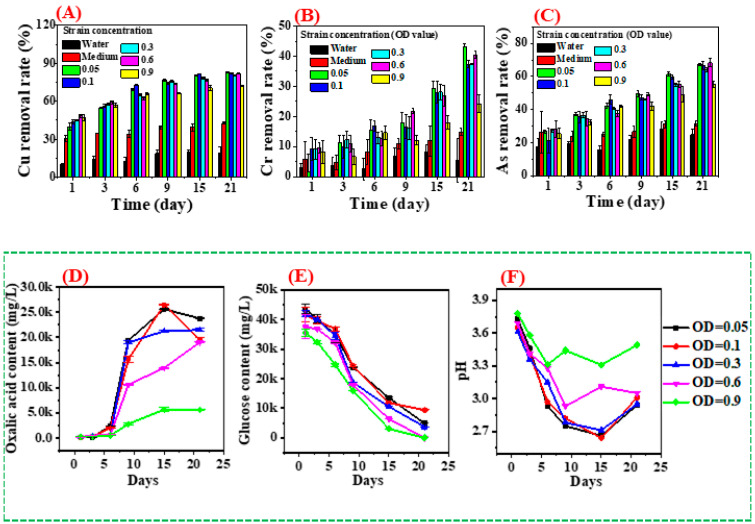
Removal rates of Cu (**A**), Cr (**B**), and As (**C**) from CCA-treated wood using *Y. lipolytica*, and the amounts of oxalic acid (**D**) and glucose (**E**) in the fermentation medium, the pH value of the fermentation medium (**F**).

The CCA-wood was treated with different concentrations of *Y. lipolytica* to remove heavy metals. An uninoculated medium and distilled water were used as controls, as shown in Figure 5A–C. After 21 days, the minimum content of copper, arsenic, and chromium in the wood was 0.417, 0.522, and 2.55 mg/g, respectively, with the treatment of yeast at OD = 0.05 concentration (Figure S1). The maximum Cu removal (82.8%) was attained after 21 days of incubation with a strain concentration of OD_600nm_ = 0.05. It was observed that almost the same amount of Cu was removed after 21 days when the strain’s concentration was OD_600nm_ = 0.1, 0.3, and 0.6. However, OD_600nm_ = 0.9 showed less Cu removal under the same condition. This was attributed to the fact that organic acids have a greater impact on metal removal than colony adsorption. In the early stage of treatment, the strain multiplies rapidly, consuming nutrients. During this period, the removal effect of heavy metals by high-concentration fungi was better than that of low-concentration fungi due to adsorption. The content of oxalic acid increased rapidly after 6 days of treatment (Figure 5C), playing a major role in removing heavy metals. The strains depleted nutrients quickly when the strains’ concentration was high. For strains with OD_600nm_ = 0.9, the lack of nutrients led to low oxalic acid production after 6 days, resulting in a low metal removal rate. This result is consistent with Table 3, where heavy metal mainly passed into the culture medium rather than the yeast, especially for low initial strains concentration. The amount of metals adsorbed by yeast increases with the initial strain concentration of the treatment solution. This increase is because the yield of organic acid is low in the initial stage of treatment, and the removal of metals is mainly by the adsorption of strains. Over time, the metal content in the yeast cells decreased. This decrease is because the organic acids produced in the late fermentation stage, especially oxalic acid, have a stronger binding ability to metals excreted from the cells to the culture solution. However, no matter the initial strain concentration, the metal content in the treated supernatant is higher than in the yeast cells. This finding suggests that metal removal is mainly due to cell secretions (organic acids) rather than the cells’ adsorption. The interactions of strains and Cu elements include extracellular complexation through organic acids, precipitation, and adsorption onto the cell wall [28]. The *Y. lipolytica* strain treatment led to extensive solubilization of Cu with increasing cultivation days under the same strains’ concentrations. It is worth mentioning that more than half of the Cu ions in the wood were removed after three days in this system. The heavy metal removal was much higher than the control samples (14.3% with water and 34.4% with medium). CCA-treated wood is impregnated with CuO, CrO_3_, and As_2_O_5_ under high pressure. These chemical compositions are water soluble, which can be leached with water or organic acids [16]. Furthermore, the medium in this study mainly consists of glucose that contains hydroxyl functional groups. These hydroxyl groups combine with heavy metals to decontaminate CCA-treated wood [16]. *Aspergillus niger,* having mature cultivation systems, has also been extensively studied in the bioremediation of treated wood. However, its removal efficiency for Cu is less than 90% after 10 days of remediation [29]. Another study showed that the Cu removal rates from CCA-treated wood treated with *Fomitopsis palustris*, *Coniophora puteana*, and *Laetiporus sulphureus* for 10 days were 71.9%, 66.5% and 50.1%, respectively [30]. Researchers have also explored other fungi to remove heavy metals from CCA-treated wood wastes, as shown in Table 4. Those results indicated the removal ability of copper ions varies with the fungal species. Those observations showed that *Y. lipolytica* is promising to obtain an excellent Cu removal rate by optimizing the medium compositions and cultivation conditions.

Compared with Cu, Cr, and As, elements in CCA-treated wood are difficult to remove. A previous study indicated that less than 20% of Cr and 30% of As were removed using *Alternaria alternata* or *Cladosporium herbarium* treated for 10 days [29]. In the current study, the removal rate of Cr at different strain concentration gradients was lower than that for Cu, where the maximum removal was 43.1%. Meanwhile, the maximum removal rate of As was 68.3%. Like Cu, the Cr and As removal rates increased with increasing incubation time (Figure 5B,C). However, Sierra-Alvarez (2009) found that the removal rate of Cu from the solid-state fermentation of CCA-treated wood by several brown rot fungi was very low (<10.9%), while a high removal rate for Cr was observed in the same system [24]. Similar results were also observed for *Fomitopsis palustris* and *Laetiporus sulphureus*, as shown in Table 4. Compared with copper ions, the removal of arsenic ions by these two fungi is more obvious. These observations are different from what has been found in this study. This observation suggests that microorganisms exhibit different tolerances to different metals. Thus, removing heavy metals from treated wood using a combination of different species of microorganisms is a promising exploration strategy. For the CCA-treated wood with uninoculated medium for 21 days, this medium was mainly composed of glucose, which contained hydroxyl structural groups. These hydroxyl groups combine with metal ions, thereby removing the metal ions from the CCA-treated wood [32].

Figure 5F shows that the pH of the medium decreased sharply before 15 days of domesticating, except for strains with OD_600nm_ = 0.6 and 0.9. The decrease in pH was accompanied by a significant increase in oxalic acid accumulation for all the samples with cultivation times up to 15 days. However, the pH values of the mediums increased from 15 to 21 days. This observation is attributed to the lack of a carbon source (i.e., glucose), leading to stagnant growth and strains’ low organic acid production. Oxalic acid in the medium complexed with copper ions further increases Cu removal under the same concentrations. Therefore, organic acids play an important role in the bioremediation process of preservative-treated wood by microorganisms [24,29]. The removal rate of heavy metals (Cu, Cr, and As) from CCA-treated wood increased with a decreased organic acid concentration in the biological system.

**Figure 6:**
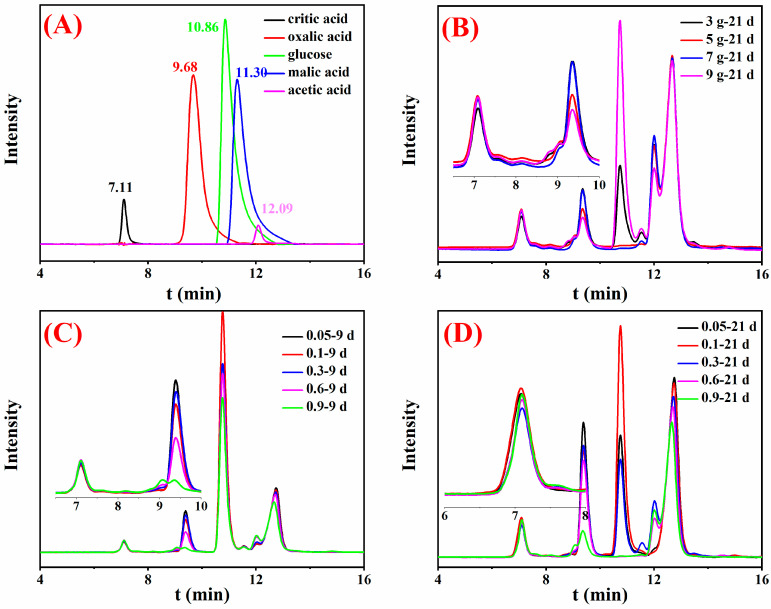
HPLC results of the standard samples (**A**) and the medium supernatant under different conditions: different contents of CCA-treated wood for 21 days (**B**), different concentrations of *Y. lipolytica* for 9 days (**C**), and different concentrations of *Y. lipolytica* for 21 days (**D**).

The organic acid composition and pH determination were analyzed to explore the metal removal mechanism for CCA-treated wood by *Y. lipolytica*. Results showed that *Y. lipolytica* produced oxalic, citric, malic, and acetic acids, as depicted in the HPLC chromatograms (Figure 6).

**Figure 7:**
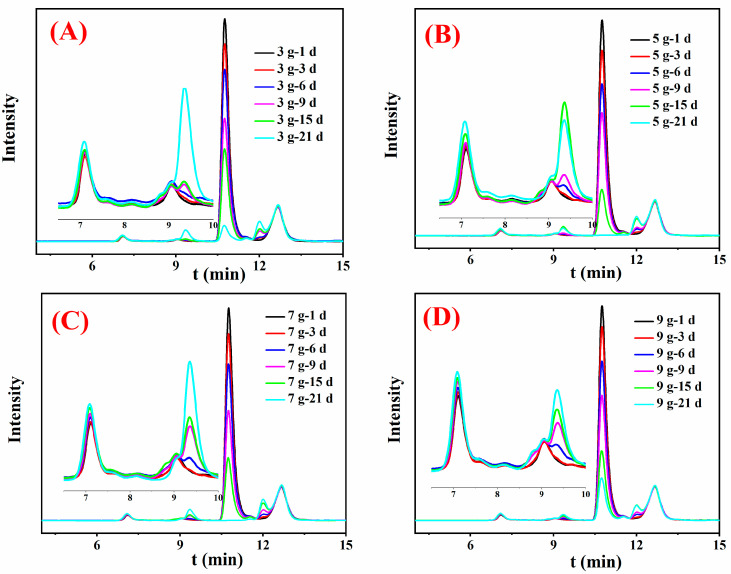
HPLC results for the medium supernatant with different contents of CCA-treated wood: CCA-treated wood of 3 g (**A**), CCA-treated wood of 5 g (**B**), CCA-treated wood of 7 g (**C**), and CCA-treated wood of 9 g (**D**).

Inoculum concentration had an important influence on the removal rate of heavy metals from the CCA-treated wood. For instance, during the first 15 days of cultivation, the removal efficiencies of Cu were shifted from low to high as follows, OD_600nm_ = 0.9 < 0.6 < 0.3 < 0.05 < 0.1. Generally, the higher the strain concentration, the increased ability to remove metal. The experimental results of this study were consistent with this rule when the treatment time was less than 6 days. However, this study showed a contrary result after 21 days of treatment. The reason is that organic acids play a major role in metal removal. In the early stage of treatment, the biosorption mechanism is dominant, so the more colonies, the more metals will be adsorbed, and the removal rate will be higher. The medium’s space and nutrients are insufficient to support the normal growth of high-concentration strains to produce oxalic acid after 6 days. The strains with low concentrations are more active and produce more contents of organic acids after 6 days, thereby possessing a higher metal removal efficiency. The removal efficiency of Cr and As was lower than that of Cu. This observation may be explained by oxalic acid being more sensitive to copper than As and Cr metals. Oxalic acid was predominant in the fermentation medium of the *Y. lipolytica* strains (Figure 6 and Figure 7). Unlike Cu, the acid that can act on chromium or arsenic was not observed. In addition, the amounts and types of organic acids produced by microorganisms vary with operating conditions, such as pH, temperature, and nutrient contents [29]. Thus, the external conditions should be optimized to adjust the removal of heavy metals from preservative-treated wood.

#### 3.3.2. Influence of Wood Amount

**Figure 8:**
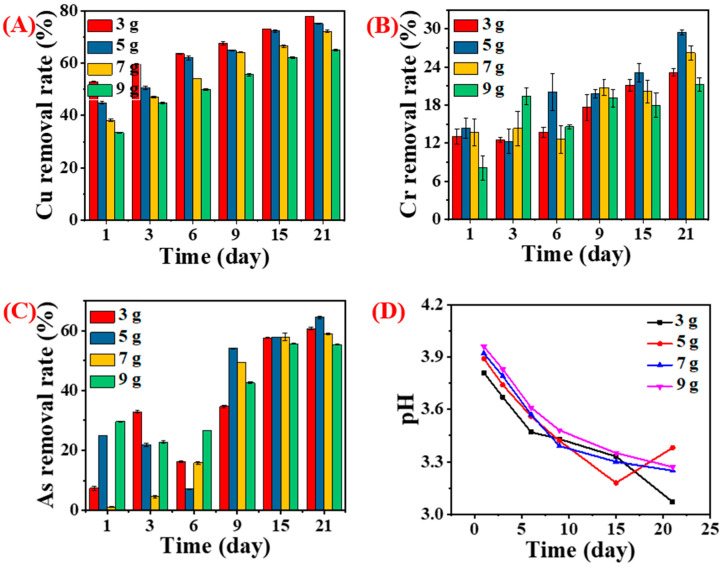
Removal rate of Cu (**A**), Cr (**B**), and As (**C**) from CCA-treated wood by *Y. lipolytica* (OD_600nm_ = 0.6) with different wood contents; pH of the fermentation medium with varying contents of CCA-treated wood (**D**).

The effect of CCA-treated wood dose on the removal of heavy metals using the liquid broth medium of *Y. lipolytica* (OD_600nm_ = 0.6) was explored. The number of wood wastes affected the removal rate of heavy metals, especially copper (Figure 8A). The percentage of removed Cu gradually decreased with increased CCA-treated wood contents (Figure 8A). Moreover, increasing the cultivation period from 1 to 21 days enhanced heavy metal removal while the pH value decreased. The pH increased with increasing wood amounts (Figure 8D). It was likely attributed to the enhancement of inorganic metal salts in liquid with the increase in CCA-treated wood dose. These observations indicated that copper removal mainly depended on the amount of organic acid. Of note, the effect of wood contents on the concentration of organic acids could have been more obvious.

The effect of wood content on the removal of Cr and As showed a different tendency compared to Cu (Figure 8B,C). The amounts of CCA-treated wood did not affect the removal rates of Cr and As. Nevertheless, they were influenced to some extent by cultivation time. In contrast, a previous study revealed that the initial concentration of ions was highly affected by removing Cr (VI) using *Y. lipolytica* in aqueous solutions [33]. In addition to the organic acids produced by the yeast, mycelium and proteins effectively decontaminated toxic metals by either biosorption or bioaccumulation [34]. Biomass residues have numerous functional groups, such as hydroxyl and carboxyl, which help absorb metal ions in aqueous solutions compared to a solid matrix. These observations reveal that the ability of yeast to decontaminate CCA-treated wood varies with metal species and the state of the pollutant.

#### 3.3.3. Influence of Copper Ion Stress

A previous study reported that the metal-tolerant properties of *Y. lipolytica* increased after strains were stressed by Cu^2+^ solutions [33]. Based on the above report, *Y. lipolytica* was domesticated by Cu ions to change its form to remove heavy metals in this study. High concentrations of Cu^2+^ were used to domesticate *Y. lipolytica* at different intervals to enhance its metal tolerance. Table 2 shows the number of colonies of *Y. lipolytica* grown on a YM solid medium domesticated by different Cu^2+^ concentrations. The number of colonies was obtained by counting. The amounts of the strains increased when the concentrations of Cu^2+^ in solution were 500 and 600 mg·L^−1^ with the increase in metal stress days. The morphology of the domesticated strains changed from large and round colonies to smaller ones. Kolhe obtained an analogous result that the dimorphic behavior of *Y. lipolytica* from yeast to mycelial forms was altered in response to metal stress [28]. In addition, biofilm formation is another way to resist metal ion stress. Moreover, the extracellular synthesis of Cu-nanoparticles of *Y. lipolytica* was also a pathway in response to Cu stress [35].

The change in morphology was significant when the strains were cultivated in a copper concentration of 700 mg·L^−1^. The number of strains first reduced within eight days and afterward increased. This can be explained by the fact that heavy metals partly inhibited the strains at an early stress stage, and the survived strains were adapted to the metal-based environment after eight days. When the concentrations of Cu^2+^ were over 800 mg·L^−1^, it was difficult for the strains to survive more than four days. Even worse, all strains died after 14 days of stress. The results indicated that the change in morphology of *Y. lipolytica* varied with concentrations of heavy metals. Similarly, previous studies found that different types of *Y. lipolytica* have differential responses to heavy metals stress [20]. A few eugenic strains were selected to treat CCA-treated wood to determine the tolerance of the domesticated strains to heavy metals.

**Figure 9:**
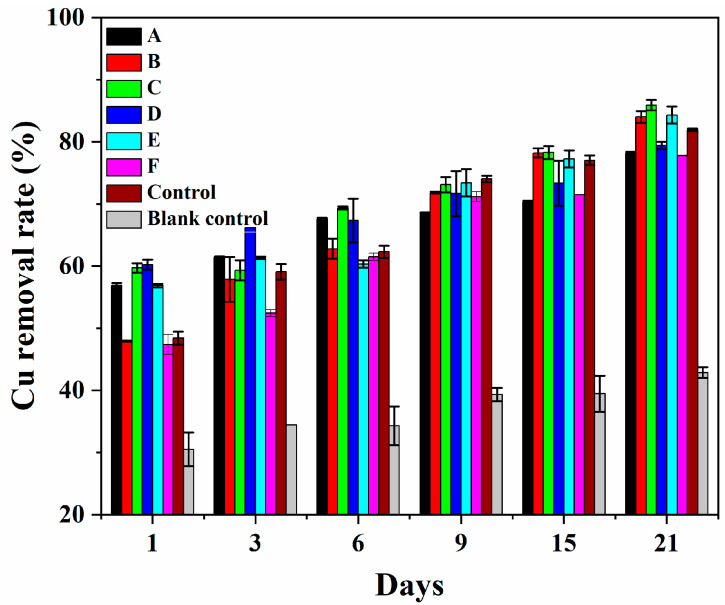
Cu removal rate from CCA-treated wood using domesticated strains compared with untreated ones; samples (A) and (B) were stressed for 4 and 8 days, respectively, with Cu^2+^ concentrations of 600 mg·L^−1^; samples (C) and (D) were stressed for 12 and 14 days, respectively, with Cu^2+^ concentrations of 700 mg·L^−1^; samples (E) and (F) were stressed for 12 days with Cu^2+^ concentrations of 800 and 1000 mg·L^−1^, respectively; the control was strains with OD_600nm_ = 0.6 without the addition of Cu^2+^; the blank control was the growth medium without any strains.

The metal stress treatment promoted the removal of copper from CCA-treated wood by *Y. lipolytica* (Figure 9). The metal adsorption performance of the seven domesticated yeasts varied with the processing parameters. The Cu^2+^ removal rate of the domesticated strains of samples (C) and (D) increased by around 20% compared to the original ones within 1 day. Meanwhile, sample C’s removal rate was 95.9% higher within one day than for the blank control (growth medium). Even though glucose and phosphate in the medium may aid in the diffusion of metals, it is difficult for them to compete for copper ions from cellulose molecules. Both glucose and wood contain a large number of hydroxyl groups. Moreover, wood contains carboxyl groups, which are easier to complex with metal ions than hydroxyl groups. The significant increase in the removal rate of copper ions indicates that *Y. lipolytica* has played an important role in promoting the removal of copper ions. Phosphatases, metallothionein, and organic acids are reported to play essential roles in overcoming copper ion stress in *Y. lipolytica* [28]. *Y. lipolytica* exhibited higher phosphatase levels in a copper-supplemented medium, which increased with metal ion concentrations [36]. The strains’ tolerance to Cu^2+^ was attributed to the increased phosphatase activity, resulting in the efflux of Cu^2+^ as phosphate complexes. The presence of phosphatase ensures the survival of fungi in the environment of high metal ions so that more organic acids can be produced to remove heavy metals from wood. Thiol groups that were in cysteine residues connected metallothionein and metal ions. Organic acids, transport proteins, and extracellular proteins secreted by the strains were imperative to the bioremediation of CCA-treated wood. They remove heavy metals by biosorption, biotransformation, bioaccumulation, and organic acid complexation [5]. Thus, the enhancement of metal removal by Cu^2+^-domesticated strains was attributed to the synergistic effect of multiple enzymes and organic acids.

The domesticated strains did not show regular growth with increasing cultivation time. The effect of sample (C) on metal removal from treated wood showed more efficiency than the original ones for different cultivation intervals. The domesticated strains (B) showed incremental patterns of metal removal with an increase in cultivation days. The metal removal rate from CCA-treated wood using strains (B) was similar to the control sample within nine days of cultivation. It overtook the control sample removal rate after 15 days of cultivation. This may be due to the original strain being adaptable to high copper ion concentrations over time. On the other hand, metal stress treatments make strains better adapt to harsh conditions than the original strain, then shorten their metal removal time.

The response of yeasts to toxic metals depends on the concentrations of heavy metal ions. Generally, yeasts adapt to the pressure of the metal environment in different ways, such as transformation, extracellular precipitation, intracellular compartmentalization, crystallization, organic acid complexation, and adsorption onto cell walls [33,37,38]. Bankar et al. (2018) investigated *Y. lipolytica’s* tolerance to heavy metals and found that the metals influenced biofilms’ formation [33]. A previous study revealed that changes in its morphological behavior demonstrated the adaption of *Y. lipolytica* to the stresses of different heavy metals [20]. In this study, strains’ morphology changed when the concentrations of Cu^2+^ were lower while the strains’ growth was inhibited at a high Cu^2+^ concentration (Table 2). Moreover, the organic acid produced by strains plays a vital role in heavy metal removal. Therefore, other stress approaches, such as changes in cultivation conditions (pH, temperature, etc.) [39], electrochemical stress [21], UV light radiation [40], and genetic engineering [41], will be further investigated to develop selective strains with better characteristics for heavy metal removal.

## 4. Conclusions

*Y. lipolytica* was efficient in the removal of toxic metals from CCA-treated wood. The copper stress of microbial strains enhanced their metal tolerance, further increasing their heavy metal removal abilities. Bioremediation changed the surface morphology and the chemicals of CCA-treated wood because heavy metals and strain residues were removed. The organic acids produced by *Y. lipolytica* played an important role in removing heavy metals, especially copper. The Cu, Cr, and As removal rates by *Y. lipolytica* were 82.8%, 43.1%, and 68.3%, respectively. Since *Y. lipolytica* does not destroy the structure of wood particles, the decontaminated CCA wood particles can be mixed with the original wood particles in a certain proportion to prepare wood pellets. In contrast to the original strains, the removal rate of copper for domesticated strains increased by 20% under fixed conditions. This result indicated that metal stress changed the nature of the strains, increasing their heavy metal removal efficiency for CCA-treated wood. This study proved that *Y. lipolytica* strains could remove metals from CCA-treated wood, advancing research on the bioremediation of solid waste pollutants.

## Figures and Tables

**Table 1:** Domesticated *Y. lipolytica* strains for bioremediation.

Sample	Concentrations of Cu^2+^ (mg·L^−1^)	Days of Metal Stress
A	600	4
B	600	8
C	700	12
D	700	14
E	800	12
F	1000	12
Control	No addition	No

**Table 2:** The number of *Yarrowia lipolytica* colonies domesticated by different Cu^2+^ concentrations grew on the YM solid medium (obtained by counting).

Concentrations of Cu^2+^	Number of Days of Metal Stress				
	1 Day	4 Days	8 Days	12 Days	14 Days
500 mg·L^−1^	116 (2.65 *)	167 (8.50)	--	--	--
600 mg·L^−1^	229 (42.77)	179 (6.51)	--	--	--
700 mg·L^−1^	170 (13.65)	92 (7.94)	21 (2.65)	99 (2.65)	--
800 mg·L^−1^	74 (14.36)	80 (6.81)	8 (3.51)	6 (1.15)	1 (0.58)
1000 mg·L^−1^	84 (15.72)	20 (2.31)	7 (2.89)	5 (3.06)	0 (0.58)

-- Uncountable, (*) standard deviation.

**Table 3:** The cu ion content in the culture supernatant and cells of *Y. lipolytica* after treatment.

	OD Values	0.05	0.6	0.6 (Fungi under Copper Ion Stress)	0.05 (Supernatant)	0.6 (Supernatant)
Time						
1 day		36.8 × 10^−3^ (mg·g^−1^)	1.53 (mg·g^−1^)	1.55 (mg·g^−1^)	46.0 × 10^−3^ (mg·g^−1^)	32.3 × 10^−3^ (mg·g^−1^)
15 days		36.8 × 10^−3^ (mg·g^−1^)	1.02 (mg·g^−1^)	1.19 (mg·g^−1^)	-	-

**Table 4:** Comparison of decontamination effects of different fungi on CCA wood.

Microbes	Maximum Metal Removal Rate (%)			Reference
	Cu	Cr	As	
*Yarrowia lipolitica*	83	43	68	This study
*Aspergillus niger*	49	55	97	[15]
*Aureobacterium barkeri*	50	68	37	[31]
*Pseudomonas fluorescens*	50	-	48	[31]
*Fomitopsis palustris*	72	87	100	[30]
*Coniophora puteana*	67	19	18	[30]
*Laetiporus sulphureus*	50	69	85	[30]

## Data Availability

The data used in this study is available upon request from the corresponding author.

## Supplementary Materials

The following supporting information can be downloaded at: https://www.mdpi.com/article/10.3390/jof9040469/s1, Figure S1: The content of copper, chromium and arsenic in wood, treated with different concentrations of yeast for different time.
