# Supplementary material for: A Comparative Study on Heavy Metal Removal from CCA-Treated Wood Waste by Yarrowia lipolytica: Effects of Metal Stress
Source: J Fungi (Basel). 2023 Apr 13;9(4):469. doi: 10.3390/jof9040469 (PMC10145133; doi:10.3390/jof9040469)
Supplement: Supplementary file 1 [file jof-09-00469-s001.zip › jof-2228452-supplementary.pdf]

Journal of *Fungi*

MDPI

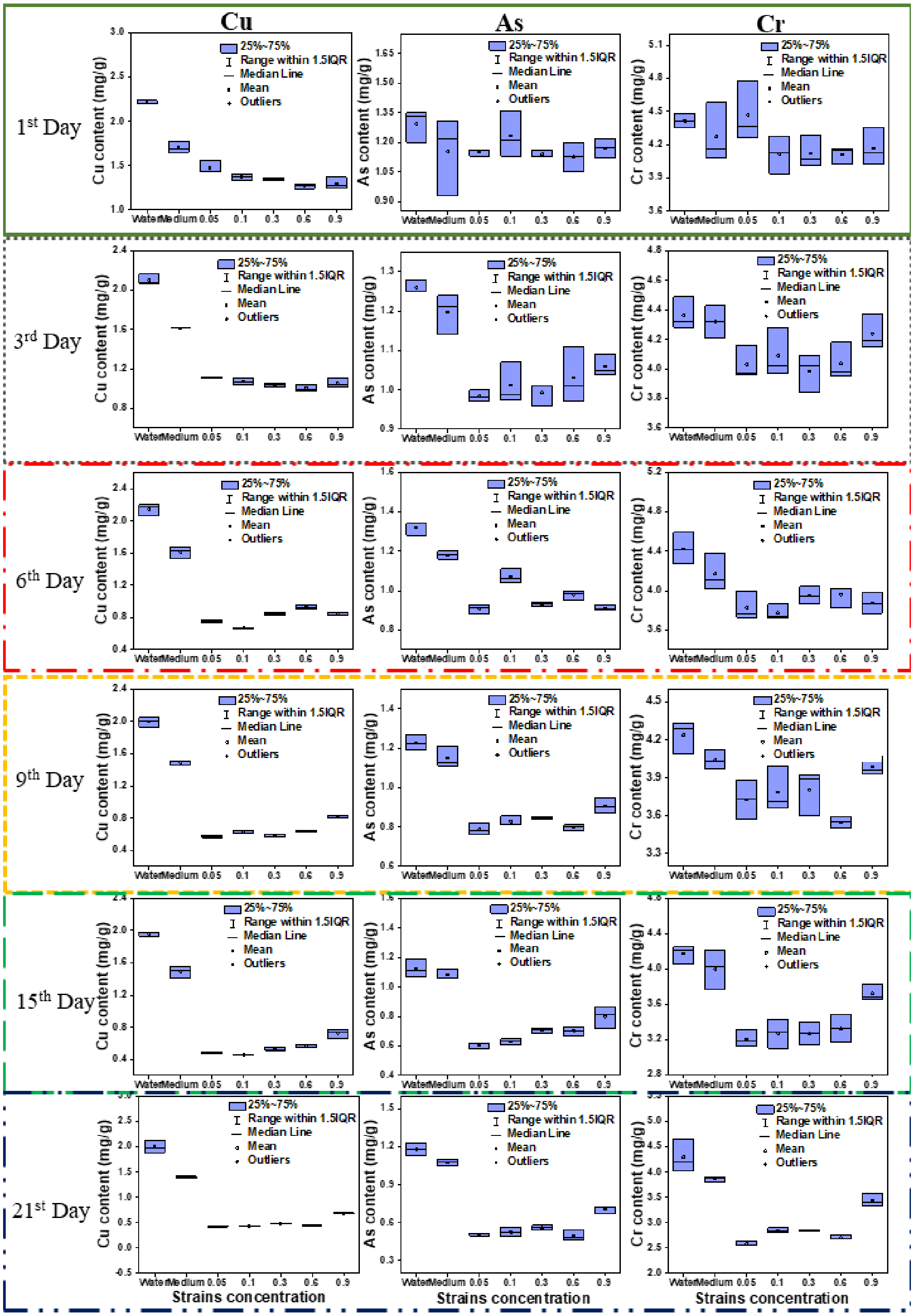

Figure S1. The content of copper, chromium and arsenic in wood, treated with different concentrations of yeast for different time.
